# Retinopathy of Prematurity in Triplets

**DOI:** 10.4274/tjo.94815

**Published:** 2016-06-06

**Authors:** Mehmet Ali Şekeroğlu, Emre Hekimoğlu, Ülker Çelik, Yusuf Kale, Ahmet Yağmur Baş

**Affiliations:** 1 Ulucanlar Eye Training and Research Hospital, Ophthalmology Clinic, Ankara, Turkey; 2 Zübeyde Hanım Maternity and Research Hospital, Ophthalmology Clinic, Ankara, Turkey; 3 Zübeyde Hanım Maternity and Research Hospital, Neonatology Clinic, Ankara, Turkey

**Keywords:** birth weight, gestational age, laser photocoagulation, retinopathy of prematurity, triplet

## Abstract

**Objectives:**

To investigate the incidence, severity and risk factors of retinopathy of prematurity (ROP) in triplets.

**Materials and Methods:**

The medical records of consecutive premature triplets who had been screened for ROP in a single maternity hospital were analyzed and presence and severity of ROP; birth weight, gender, gestational age of the infant; route of delivery and the mode of conception were recorded.

**Results:**

A total of 54 triplets (40 males, 14 females) who were screened for ROP between March 2010 and February 2013 were recruited for the study. All triplets were delivered by Caesarean section and 36 (66.7%) were born following an assisted conception. During follow-up, seven (13%) of the infants developed ROP of any stage and two (3.7%) required laser photocoagulation. The mean gestational age of triplets with ROP was 27.6±1.5 (27-31) weeks whereas it was 32.0±1.5 (30-34) weeks in those without ROP (p=0.002). The mean birth weights of triplets with and without ROP were 1290.0±295.2 (970-1600) g and 1667.5±222.2 (1130-1960) g, respectively (p<0.001). The presence of ROP was not associated with gender (p=0.358) or mode of conception (p=0.674).

**Conclusion:**

ROP in triplets seems to be mainly related to low gestational age and low birth weight. Further prospective randomized studies are necessary to demonstrate risk factors of ROP in triplets and to determine if and how gemelarity plays a role in the development of ROP.

## INTRODUCTION

Retinopathy of prematurity (ROP) is one of the major causes of preventable childhood blindness.^1^ Since the most significant risk factors are low birth weight and low gestational age, the number of infants at risk for ROP started to increase after the improvement of survival of extremely premature infants.^2^ Other proposed risk factors for ROP include respiratory distress syndrome, prolonged mechanical ventilation, blood transfusion, sepsis, assisted conception and multiple births.^3,4,5,6^ However, the association between multiple births and the incidence and severity of ROP is still contradictory.^7,8^ The aim of the present study was to assess the incidence, severity and risk factors of ROP in triplets.

## MATERIALS AND METHODS

This retrospective study was carried out in a single maternity and research hospital in full accord with the principles laid out in the Declaration of Helsinki, upon approval of Institutional Review Board. The data was obtained by chart review. The medical files of premature infants admitted for routine ROP screening examination were reviewed and consecutive triplets who were followed for at least 6 months were recruited to the study. The rendered data included the presence and severity of ROP; birth weight, gender, gestational age of the infant; route of delivery and the mode of conception (assisted or natural conception). The timing of the initial screening examination, frequency of follow-up examinations and when to discontinue screening examinations was determined according to the recommendations of the American Academy of Pediatrics, American Academy of Ophthalmology, and American Association for Pediatric Ophthalmology and Strabismus.^9^ Screening was performed by one of two experienced pediatric ophthalmologists (MAŞ and EH). Prior to examination, pupillary dilation was induced with 2.5% phenylephrine and 0.5% tropicamide, after which the topical anesthetic proparacaine was instilled and an eyelid speculum was applied. Each infant underwent a binocular indirect ophthalmoscopy examination for each eye with a 28 D condensing lens and scleral depression. Treatment decisions were made according to ‘The Early Treatment of Retinopathy of Prematurity study’ criteria.10 Statistical analysis was performed using SPSS software for Windows 15.0 (Statistical Package for the Social Sciences, SPSS, Inc., Chicago, IL, USA). Median and range were given as descriptive statistics for quantitative data. Categorical data were summarized using frequency and percentages. Independent samples t test was used to compare two independent groups with normal distribution of quantitative data and Mann-Whitney U test was used for abnormal distributions. Results were accepted as statistically significant when p was <0.05.

## RESULTS

A total of 54 triplets (40 males, 14 females) who were screened for ROP between March 2010 and February 2013 were included in the study. Gestational age of the infants at birth ranged from 27 to 34 weeks with a mean gestational age of 31.4±2.1 weeks. Birth weight ranged from 970 to 1.960 g with a mean of 1.618.5±262.9 g. All triplets were delivered by Caesarean section and 36 (66.7%) were born following an assisted conception. Seven (13%) of the infants developed ROP of any stage and two (3.7%) required transpupillary diode laser photocoagulation during follow-up. The two infants who required laser treatment were siblings. Their third sibling had zone II stage 2 ROP which spontaneously regressed without any treatment. Infants requiring treatment for ROP were those with the lowest gestational age (27 weeks) and lowest birth weight (970 and 980 g) among the whole study population. The distribution of ROP according to gestational age groups are shown in Table 1. The mean gestational age of triplets with ROP was 27.6±1.5 (27-31) weeks and 32.0±1.5 (30-34) weeks in those without ROP (p=0.002). The mean birth weight of triplets with ROP was 1,290.0±295.2 (970-1,600) g compared to 1,667.5±222.2 (1,130-1,960) g in those without ROP (p<0.001). The presence of ROP was not associated with gender (p=0.358) or mode of conception (p=0.674).

## DISCUSSION

The incidence of multiples pregnancies has increased, possibly because of higher maternal age and the increased use of assisted conception methods such as ovulation induction and in vitro fertilization.^11^ These are known to be associated with maternal and neonatal complications which are directly related to the number of fetuses in utero. A large study reported that 15% of singletons, 48% of twins and 78% of higher order multiples require neonatal intensive care unit (NICU) admission.^12^ The higher rate of neonatal complications is mostly due to prematurity. However, the relationship between multiple pregnancies and ROP is still controversial.^7,8,13^ Some investigators assume multiple gestation is an independent risk factor for ROP,^14,15^ but others do not.^13,16^

Triplet birth is a rare occurrence. The incidence of triplets increased after the introduction of assisted conception methods, but started to decline again after limitation of the number of embryos transferred.^17^ In a study investigating NICU admissions, the proportion of triplets reported to be decreased from 5.0 to 3.3 per 100 NICU admissions.^18^ Preterm labor and prematurity were the commonest complications of triplet gestations. Chibber et al.^19^ reported the rate of prematurity as 84% in their large triplet series. In a study investigating fetomaternal outcome in triplet pregnancy, mean birth weights of 1st, 2nd and 3rd triplets were 1.651, 1.640 and 1.443 g respectively.^20^ Zanconato^21^ reported the incidence of preterm labor as 78.6% and ROP as 6.5% in triplet pregnancies. The incidence of any stage of ROP was 13% and the rate of ROP requiring treatment was 3.7% in our series.

The relationship between triplet pregnancies and ROP is also controversial. Tomazzoli et al.^8^ found no increased risk for ROP when they compared premature triplets with premature singletons, and concluded that multiple gestation adds no risk beyond that due to prematurity. Assisted conception methods also did not appear to be an independent risk factor for ROP in our study, similar to previous studies in the literature.^7,17,19^ Nevertheless, gynecologists should be aware of possible neonatal outcomes of triplet pregnancies and the higher risk of prematurity in order to follow proper infertility management strategies and avoid iatrogenic multiple gestations.

We evaluated triplets for certain clinical and demographical features including birth weight, gestational age, gender, route of delivery and mode of conception, and found birth weight and gestational age were related to ROP. The two siblings who required treatment for ROP in our study population were extremely premature (with the lowest gestational age and birth weights of the study population). Gender, route of delivery and mode of conception were not associated with ROP. Maayan-Metzger et al.^16^ compared triplet and singleton preterm infants and found no differences between two groups in terms of perinatal parameters, respiratory parameters and neonatal complications, including ROP. We did not investigate some other proposed risk factors such as respiratory distress syndrome, septicemia, number of blood transfusions and duration of mechanical ventilation.

The present study should be viewed in context of some limitations. First of all, the limited number of triplets may influence the power of statistical outcomes. Regression analysis for risk factors could not be performed because of the small number of infants with ROP. Secondly, other ROP risk factors such as respiratory distress syndrome, duration of mechanical ventilation, septicemia, and blood transfusion were not included in the study parameters. Thirdly, the study was retrospective, which limited the data available. Finally, there was a lack of control group such as singleton or twin gestations for comparing outcome data. Despite these limitations, the current study has important implications regarding the incidence, severity and risk factors of ROP in triplets. In light of the present data, as the most important risk factors are low birth weight and low gestational age for ROP in triplets, we can suggest that an ROP screening protocol similar to that used for singletons can be applied in triplets.

## CONCLUSION

Triplet pregnancies usually result in prematurity. ROP in triplet infants seems to be mainly related to low gestational age and low birth weight. Larger prospective randomized studies are necessary to demonstrate risk factors of ROP in triplets and to determine if and how gemelarity plays a role in the occurrence of ROP.

## Ethics

Ethics Committee Approval: The study was approved by the Zübeyde Hanım Gynecology Training and Research Hospital of Local Ethics Committee, Informed Consent: Consent form was filled out by all participants.

Peer-review: Externally peer-reviewed.

## Figures and Tables

**Table 1:**
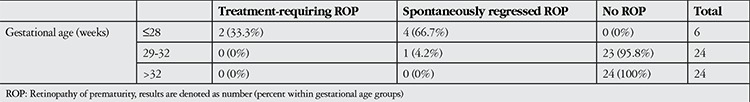
The distribution of retinopathy of prematurity according to gestational age groups in triplets
